# Supplementary figures and images for: Caenorhabditis elegans DBL-1/BMP Regulates Lipid Accumulation via Interaction with Insulin Signaling
Source: G3 (Bethesda). 2017 Nov 21;8(1):343–51. doi: 10.1534/g3.117.300416 (PMC5765361; doi:10.1534/g3.117.300416)

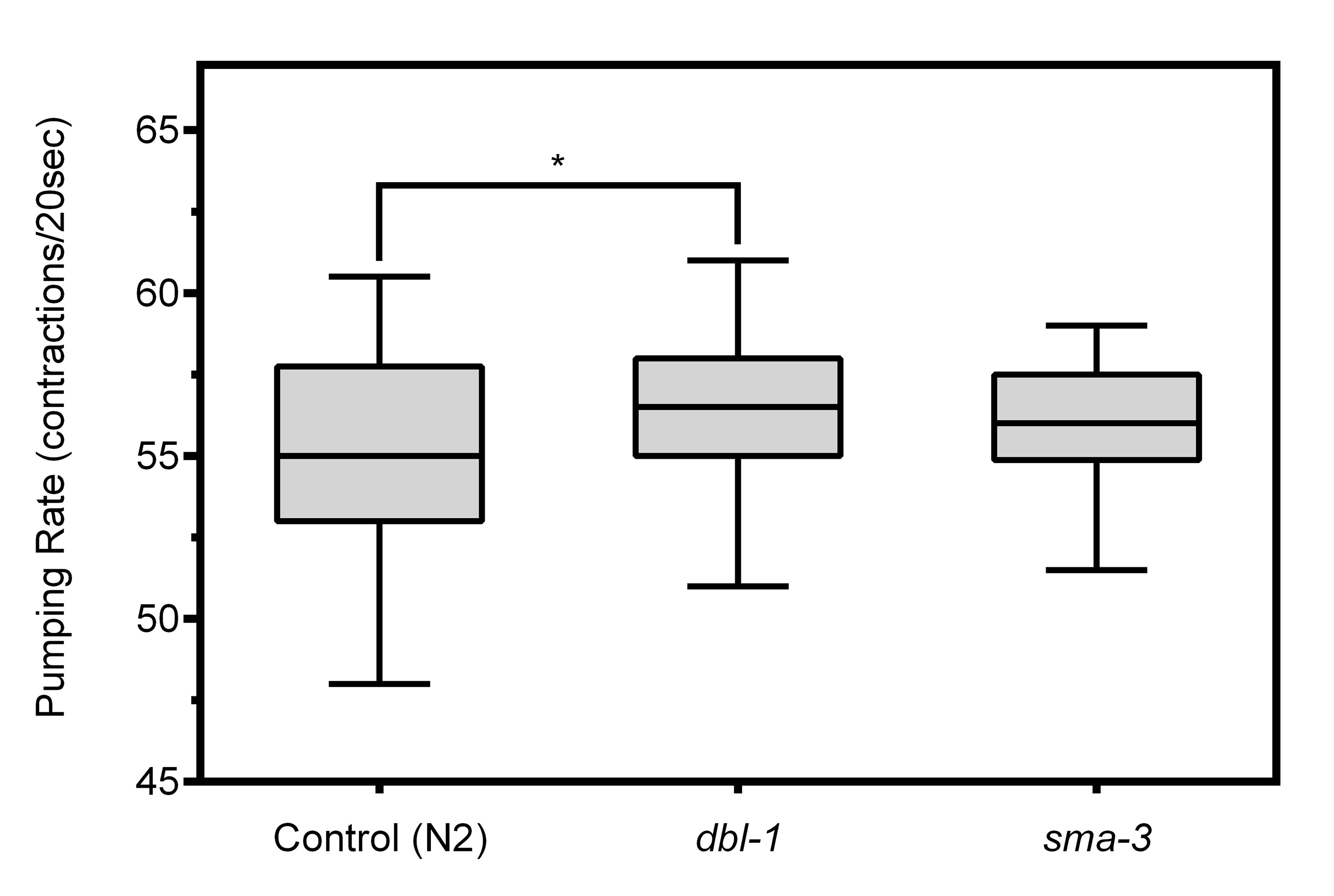

Supplement: Supplementary file 1 [file 343FigureS1.tif]

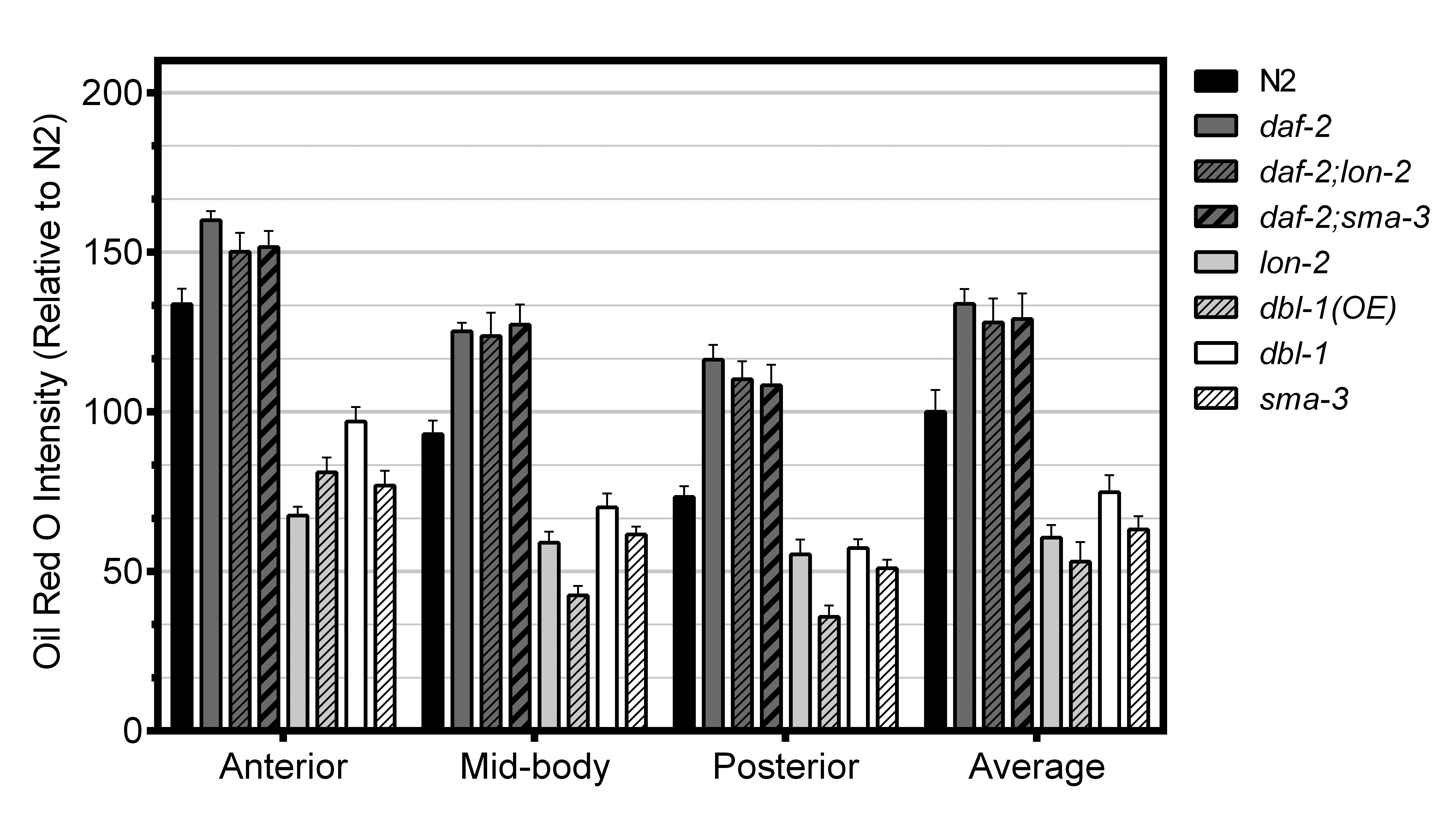

Supplement: Supplementary file 2 [file 343FigureS2.tif]
